# Evaluation of recombinase polymerase amplification for detection of begomoviruses by plant diagnostic clinics

**DOI:** 10.1186/s12985-016-0504-8

**Published:** 2016-03-22

**Authors:** Maria A. Londoño, Carrie L. Harmon, Jane E. Polston

**Affiliations:** Department of Plant Pathology, University of Florida, Gainesville, FL 32611 USA

**Keywords:** Plant virus detection, Plant pathogen diagnostic assay, Polymerase chain reaction, Recombinase polymerase amplification

## Abstract

**Background:**

Plant viruses in the genus *Begomovirus*, family *Geminiviridae* often cause substantial crop losses. These viruses have been emerging in many locations throughout the tropics and subtropics. Like many plant viruses, they are often not recognized by plant diagnostic clinics due in large part to the lack of rapid and cost effective assays. An isothermal amplification assay, Recombinase polymerase amplification (RPA), was evaluated for its ability to detect three begomoviruses and for its suitability for use in plant diagnostic clinics. Methods for DNA extraction and separation of amplicons from proteins used in the assay were modified and compared to RPA manufacturer’s protocols. The modified RPA assays were compared to PCR assays for sensitivity, use in downstream applications, cost, and speed.

**Results:**

Recombinase polymerase amplification (RPA) assays for the detection of *Bean golden yellow mosaic virus*, *Tomato mottle virus* and *Tomato yellow leaf curl virus* (TYLCV) were specific, only amplifying the target viruses in three different host species. RPA was able to detect the target virus when the template was in a crude extract generated using a simple inexpensive extraction method, while PCR was not. Separation of RPA-generated amplicons from DNA-binding proteins could be accomplished by several methods, all of which were faster and less expensive than that recommended by the manufacturer. Use of these modifications resulted in an RPA assay that was faster than PCR but with a similar reagent cost. This modified RPA was the more cost effective assay when labor is added to the cost since RPA can be performed much faster than PCR. RPA had a sensitivity approximate to that of ELISA when crude extract was used as template. RPA-generated amplicons could be used in downstream applications (TA cloning, digestion with a restriction endonuclease, direct sequencing) similar to PCR but unlike some other isothermal reactions.

**Conclusions:**

RPA could prove useful for the cost effective detection of plant viruses by plant diagnostic clinics. It can be performed in one hour or less with a reagent cost similar to that of PCR but with a lower labor cost, and with an acceptable level of sensitivity and specificity.

**Electronic supplementary material:**

The online version of this article (doi:10.1186/s12985-016-0504-8) contains supplementary material, which is available to authorized users.

## Background

Rapid, sensitive and specific detection of plant pathogens is an important aspect of disease management. Polymerase chain reaction (PCR) has become one of the most commonly-used nucleic acid based methods for the detection of plant pathogens due to its speed, specificity and sensitivity [1]. PCR and reverse-transcriptase PCR (RT-PCR) can be designed to detect a narrow or broad range of targets through the use of specific or degenerate primers and amplified products can be viewed in electrophoretic gels, sequenced directly, and/or cloned. [2–4]. The disadvantages of PCR/RT-PCR for pathogen detection are the dependence on a thermocycler, inhibitory effects of co-extracted host plant inhibitors on amplification, and the time investment per sample [5–7]. While PCR/RT-PCR assays are used in the detection of plant viruses in research, the cost and time required are too great for many plant disease clinics. Real-time PCR (qPCR), reduces the time required for detection compared to PCR/RT-PCR, but has high start-up and instrument maintenance costs which has discouraged its adoption by many plant disease clinics.

Some of the disadvantages of PCR/RT-PCR have been avoided by the use of loop mediated isothermal amplification (LAMP) assays, which have been developed for over 100 animal and plant pathogens [8–10]. LAMP is an isothermal reaction that has a sensitivity and specificity similar to that of PCR. LAMP does not require a thermocycler, uses a shorter amplification time than PCR, but like PCR/RT-PCR requires a RNA or DNA template mostly free of host contaminants. However, downstream applications of LAMP products such as direct sequencing, cloning and restriction analysis are more complicated than PCR/RT-PCR, as LAMP generates multimeric products [11].

Another type of isothermal amplification methodology is Recombinase Polymerase Amplification (RPA). RPA was first developed in 2006 and relies on the extension of primers induced by recombination proteins [12]. DNA binding proteins (gp32 single-strand DNA binding protein and two ATP-dependent recombinases, usvX and usvY) bind the primers and scan for the homologous sequence (target). The primers recombine with the target, and a mesophilic polymerase (*Bacillus subtilis* DNA polymerase I) extend the 3’ end of the invading primer using the opposite strand as a template [12]. RPA reactions have been performed at constant low temperatures (25 °C to 42 °C), achieving amplification in as little as 15 min. PCR purification columns remove DNA-binding proteins from the amplicons to allow visualization of results. The use of columns adds to the expense and time required to complete the assay. The most commonly used methods to obtain results of RPA assays are visualization of amplified DNA by gel electrophoresis or amplicon sequencing although alternatives such as fluorescence and/or hybridization have been reported [12–14]. In addition to end-point detection of DNA targets, RPA formats have been developed for detection of RNA templates (RT-RPA), target quantification and chip-based detection [13–16], which demonstrate the flexibility of this type of assay for rapid pathogen diagnostics. RPA is widely used in the detection of animal and human pathogens [13, 17]. Its use in plant pathogen detection has been limited to four plant pathogens: two viruses, (*Little cherry virus 2* (LChV2) and *Plum pox virus* (PPV)), a bacteria (*Candidatus* Liberibacter asiaticus (HLB - Las)) and a fungus (*Fusarium oxysporum* f.sp. *vasinfectum*) ([18, 19] AgDia, Inc. Elkhart, IN).

There is a real need in diagnostic laboratories for technology that provides rapid and affordable diagnosis of viruses and has the flexibility for downstream applications or applied disease management. This is especially true for emerging viruses such as species in the genus *Begomovirus* (Family *Geminiviridae*) [20–23]. Species in the genus have been emerging over the last three decades to become plant pathogens that threaten crop production in tropical and subtropical regions around the world. One of these viruses, *Tomato yellow leaf curl virus* (TYLCV), emerged in the eastern Mediterranean in the 1960s. TYLCV has since spread via the plant trade to virtually all tomato production areas wherever its vector, *Bemisia tabaci* species complex, is endemic [24]. Current methods for the detection of TYLCV and other begomoviruses are primarily PCR, but other methods include enzyme-linked immunosorbent assay (ELISA), lateral flow immunochromatographic assays, dot blot hybridization, rolling circle amplification (RCA) and LAMP [25–33].

This manuscript describes the development and diagnostic laboratory evaluation of RPA for the detection of three begomoviruses. We modified RPA assays to reduce expense and time, and compared these with RPA assays using manufacture’s recommended protocols. We then compared RPA with the better known PCR in terms of sensitivity, specificity and adaptation to downstream applications in the detection of TYLCV.

## Methods

### Virus-infected plant material

TYLCV (GenBank:AY530931) was maintained in tomato (*Solanum lycopersicum* L. ‘Florida Lanai’) in a plant growth room by whitefly transmission [34]. Tobacco (*Nicotiana tabacum* L. ‘Samsun’) and bean (*Phaseolus vulgaris* L. ‘Top Crop’) plants were infected with TYLCV using whiteflies from infected tomatoes [34]. Bean seedlings were infected with *Bean golden yellow mosaic virus* (BGYMV, GenBank:DQ119824, DQ11982425), *Euphorbia mosaic virus* (EuMV, GenBank:KJ647290, KJ647291), *Sida golden mottle virus* (SiGMoV, Genbank:GU997691, GU997692) and *Tomato mottle virus* (ToMoV, GenBank:L14460, L14461) by biolistic inoculation using infectious clones [31]. In addition, TYLCV-inoculated bean and tomato leaf tissues stored at −20 °C for 5 months, −80 °C for 3 months, or desiccated and stored at 4 °C for approximately 15 years were included in the evaluation.

### Template preparation

DNA (“purified DNA”) was extracted from fresh, frozen and desiccated leaves of *P. vulgaris*, *N. tabacum* and *S. lycopersicum* using Puregene DNA reagents (Qiagen, Valencia, CA) and the following protocol: leaf tissue (10–30 mg) was ground in liquid nitrogen then suspended with 300 μl of Cell Lysis Solution. The lysate was incubated at 65 °C for 60 min and cooled to room temperature for 5 min, after which 100 μl of Protein Precipitation Solution was added. Samples were briefly vortexed and incubated on ice for 10 min after which they were centrifuged at 20,000 rcf for 10 min. The supernatant was collected and nucleic acids were precipitated using 300 μl of isopropanol and centrifuged at 18,000–20,000 rcf for 5 min. The pellet was then washed with 70 % ethanol and resuspended in 50 μl of DNA Hydration Solution. Crude extracts were prepared in 0.5 N NaOH from fresh, frozen and desiccated material using a technique modified from Satya et al. 2013 [6]. Fresh or frozen leaves (10 mg) were homogenized using a plastic pestle in a 2 ml microfuge tube with 30 μl 0.5 N NaOH. Dessicated leaf tissues were homogenized in the same way but using 0.5 N NaOH at 1:18 w/v. Crude extracts were centrifuged at 20,800 rcf for 3 s to separate out particulate matter and the supernatant was collected for use as template.

TYLCV DNA cloned in pBluescript KS- at an *Xba*I site was purified from *E. coli* using NucleoSpin® Plasmid kit (Macherey-Nagel GmbH & Co, Düren, Germany). The concentration of DNA (39.74 ng/μl) was determined using a Nanodrop 2000 (Thermo Scientific, Wilmington DE) and calculating the mean of three repeats for each treatment. The concentration of TYLCV DNA (19.87 ng/μl), was determined in the undiluted aliquot by multiplying the total amount of DNA by 48.3 % which represented the percent of TYLCV DNA in the total extracted DNA (insert plus plasmid). Tenfold dilutions of that aliquot were made using nuclease-free water.

### Primer design

All primers (Table 1) were designed to function in both PCR and RPA. As recommended for RPA, primers were ≥ 29 bp long, were GC-rich in the 3’-end, contained a pyrimidine at the 5’-end, and amplified fragments smaller than 500 bp [12, 13]. Two primers were modified from existing primers TYC1F and TYC1R which had been used in PCR to amplify a portion of the C1 gene of TYLCV [32]. Primer TYC1F (underlined) was expanded to include more of the TYLCV genome sequence to create TYL828F: 5’-CCTAGAGACCTGGCCCACATTGTTTTGCCTGTTCTGC-3’, and similarly, TYC1R (underlined) was expanded to create primer TYL832R 5’-CCATCCGGTAATATT ATACGGATGGCCGC-3’.

**Table 1:** Primers used in these studies

Target virus	Primer pair (nt positions in genome)	Sequence 5′-3′	Expected amplicon length (nt)
BGYMV	BGY1141F (2447–2479)	TTGGACTGAACTCTAAATCTTTGAGGTTGTGGC	330
	BGY1142R (130–97)	CCAAGTTTTACGAATATCTAGGCTCGTCAAACGC	
ToMoV	ToMo1131F (1073–1109)	TGATCCAGAAGCTGTCATCGACGTCGTCCAAACTTGG	325
	ToMo1139R (1397–1364)	CAAGCCAAGAAACGGGCAATCAGAAGGCGCAGGG	
TYLCV	TYL830F (2345–2381)	CCTGAATGTTCGGATGGAAATGTGCTGACCTGCTTGG	446
	TYL832R (10–2762)	CCATCCGGTAATATTATACGGATGGCCGC	
TYLCV	TYL828F (1918–1954)	CCTAGAGACCTGGCCCACATTGTTTTGCCTGTTCTGC	464
	TYL834R (2380–2346)	TCCAAGCAGGTCAGCACATTTCCATCCGAACATTCGG	

### PCR assays

All PCR reaction mixtures contained the following: 10 mM Tris–HCl, 50 mM KCl, 2.5 mM MgCl_2_, 0.20 μM primer (each), 0.1 mM dNTPs, 1 mM spermidine, 0.625 units of *Taq* DNA polymerase (New England Biolabs, Ipswich, MA, USA) and 0.5 μl of template for a total volume of 25 μl. All amplification cycles used an initial denaturation at 95 °C for two min and a final extension at 72 °C for seven min. Amplification cycles were as follows: TYLCV primer pairs TYL828F/TYL834R and TYL830F/TYL832R: 35 cycles of 95 °C for 30 s, 60 °C for 30 s, and 72 °C for 30 s; ToMoV primer pair ToMo1131F/ToMo1139R and BGYMV primer pair BGY1141F/BGY1142R: 35 cycles of 95 °C for 30 s, 60 °C for 30 s, and 72 °C for 20 s. PCR amplicons were visualized by the addition of ten microliters amplified DNA to 1.5 % agarose gels containing ethidium bromide.

### RPA assays

RPA assays were performed using reagents in the Twist Amp® Basic kit (TwistDx Limited, Cambridge, UK) in 50 μl total volume according to the manufacturer’s instructions. The reaction mix and the template (0.5 μl) were added to the corresponding reaction pellet tube, followed by 2.5 μl of 280 mM magnesium acetate. Reactions were incubated at 37 °C for 30 min using a water bath, heating block or thermocycler. Proteins were separated from the amplified DNA (“cleaned”) with either QIAquick PCR purification columns (Qiagen, Valencia, CA) or using one of the methods described below. Unless otherwise stated, RPA products were denatured at 65 °C for 10 min before 10 μl were loaded into wells of 1.5 % agarose gels containing ethidium bromide.

### Visualization of RPA amplicons

The manufacturer recommends that amplicons generated by TwistAmp® Basic be cleaned of DNA-binding proteins using PCR purification columns in order to be visualized using agarose gel electrophoresis [12, 14]. Since PCR purification columns increase the expense of an RPA-based detection assay, we compared RPA amplicons cleaned with PCR purification columns with those cleaned by less expensive methods that would be expected to denature proteins and separate them from amplified DNA (heating, addition of sodium dodecyl sulfate (SDS) or formamide).

DNA extracted from TYLCV-infected tomato leaves was amplified by RPA using primer pair TYL828F/TYL834R. The amplicons were cleaned using one of these methods: QIAquick columns according to the manufacturer’s instructions (Qiagen, Valencia, CA); incubation at 65 °C for 10 min; incubation at 95 °C for 10 min; addition of SDS to the loading buffer to a final concentration of 5 % w/v; addition of SDS to a final concentration of 10 % w/v %; or addition of formamide to a final concentration of 15 %. All treatments were repeated three times.

### Comparison of sensitivity of detection by RPA with PCR

The sensitivity of RPA was compared to that of PCR using serial dilutions of three sources of TYLCV DNA (cloned, purified from plants and crude extracts) and primer pair TYL828F/TYL834R. Each template was diluted serially up to 10^9^-fold and each diluted template was evaluated using PCR and RPA. DNA purified from cloned TYLCV DNA was diluted with water, DNA purified from TYLCV-infected plants was diluted using DNA purified from non-inoculated plants, and DNA in crude extracts was diluted using crude extracts from non-inoculated plants. The PCR and RPA reactions were conducted as described. RPA amplified DNA was cleaned using a column with a final elution volume of 30 μl, of which ten microliters was added to agarose gels for visualization.

### Downstream applications

Amplicons generated by RPA using either purified DNA or crude extracts were evaluated and compared for their ability to be used in three downstream applications: TA cloning, restriction endonuclease digestion and direct sequencing. RPA amplicons were generated using primer pair TYL828F/TYL834R for 20 min at 37 °C. Amplicons were treated by one of four methods before use in downstream applications: QIAquick columns, incubation at 65 °C for 10 min followed by centrifugation at 3800 rcf for 20 s, incubation at 65 °C for 10 min, or no post-amplification treatment. The efficiency of TA cloning was defined as the number of colonies with the expected insert in ten randomly selected white colonies. One clone from each treatment was sequenced to confirm identity. Restriction endonuclease digestion consisted of digestion of amplicons with *Cla*I (New England Biolabs, Ipswich, MA, USA) for 1 h at 37 C and results were visualized in 1.5 % agarose gels. Cloning of amplicons used pGEM-T-Easy (Promega, Madison, WI, USA) and *E. coli* JM109 competent cells (Promega, Madison, WI, USA). Direct sequencing was attempted using RPA amplicons generated from crude extracts and from DNA purified from two TYLCV-infected tomato plants. Direct sequencing was attempted from amplicons cleaned using QIAquick columns or incubation at 65 °C for 15 min followed by centrifugation at 3800 rcf for 20 s. Amplicons were sent to Eurofins Genomics (Huntsville, AL) for sequencing using primer pair TYL828F/TYL834R.

### Clinical trial

The UF Plant Diagnostic Center received 13 field samples from suspected TYLCV-infected tomato plants from two locations. These samples were tested for the presence of TYLCV using RPA and PCR. The PCR protocol was performed using DNA extracted with the DNeasy plant mini kit (Qiagen, Valencia, CA), amplified with primer pair TYL828F/TYL834R using ReadyMix™ Taq PCR Reaction Mix (Sigma-Aldrich, St Louis, MO). The RPA protocol used a crude extract template, amplification using primer pair TYL828F/TYL834R for 20 min at 37 °C, and cleaning of amplicons by incubation at 65 °C for 10 min. The amplifications by both RPA and PCR were repeated.

### Comparison of cost and time

The costs of RPA- and PCR-generated amplicons were compared using prices in US dollars (USD) current as of June 2015 for reagents that were used in the clinical trial at the UF Plant Diagnostic Clinic. The time required to obtain results from RPA and PCR assays were recorded using assays for single samples as well as multiple samples.

## Results

### Evaluation of primers for detection of begomoviruses by RPA

**Fig. 1:**
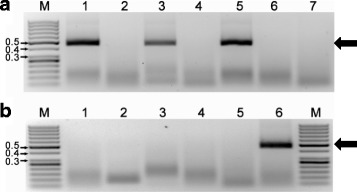
Detection of *Tomato yellow leaf curl virus* (TYLCV) by RPA and purified DNA as template. Amplification using primer pair TYL828F/TYL834R from different host plants **a** Lane M: 50 bp ladder MW standard, size is indicated in kilobases (kb); Lane 1 TYLCV-infected tomato; Lane 2 non-inoculated tomato; Lane 3 TYLCV -infected bean; Lane 4 non-inoculated bean; Lane 5 TYLCV-infected tobacco; Lane 6 non-inoculated tobacco; Lane 7 water control. Amplification from *P. vulgaris* (bean) infected with one of four different begomoviruses **b** Lane 1 non-inoculated, Lane 2 *Bean golden yellow mosaic virus* (BGYMV); Lane 3 *Euphorbia mosaic virus* (EuMV); Lane 4 *Sida golden mottle virus* (SiGMoV); Lane 5 *Tomato mottle virus* (ToMoV); Lane 6 TYLCV. Ten μl of amplified product (cleaned by heating to 65 °C for 10 min) were loaded into each lane of the 1.5 % agarose gels and stained with ethidium bromide

RPA, using primer pair TYL828F/TYL834R, generated a single amplicon band of 464 bp from TYLCV-infected leaf tissues in all three host plants (tomato, bean, tobacco). Only a very low amplification background was present in non-inoculated tissues of the same host species (Fig. 1a). No amplicons of the expected size were generated from DNA extracted from non-inoculated plants. The same primer pair did not amplify the expected size amplicon in DNA extracted from bean plants infected with BGYMV, EuMV, SiGMoV or ToMoV (Fig. 1b). Similar results were observed with RPA and primer pairs, BGY1141F/BGY1142R and ToMo1131F/ToMo1139R, designed to generate amplicons from BGYMV and ToMoV, respectively (Additional file 1: Figure S1). The identity of the amplicons generated by all three primer pairs were confirmed by direct sequencing. The condition of the tissue (fresh, frozen or desiccated) did not appear to have an impact on amplification. Results with primer pair TYL828F/TYL834R are shown (Fig. 1a). Similarly, RPA and primer pairs BGY1141F/BGY1142R and ToMo1131F/ToMo1139R generated the expected size amplicons regardless of whether the DNA was extracted from fresh leaf tissue or from leaves stored at −20 °C (data not shown). All primer pairs tested generated amplicons of the expected sizes by PCR and by RPA.

### Visualization of RPA amplicons

**Fig. 2:**
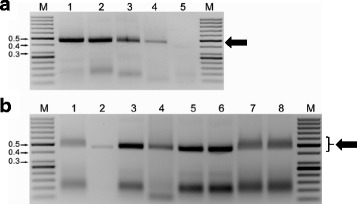
Comparison of selected treatments on the visualization of RPA-generated amplicons. **a** Results using primer pair TYL828F/TYL834R from crude extracts prepared in 0.5 N NaOH from *Tomato yellow leaf curl virus* (TYLCV)-infected tomato leaves and cleaned by heating to 65 °C for 15 min. Lane M: 50 bp ladder MW standard, size is indicated in kilobases (kb); Lane 1 fresh tissue; Lane 2 tissue kept frozen at −20 °C for 5 months; Lane 3 tissue kept frozen at −80 °C for 3 months; Lane 4 desiccated tissue maintained at room temperature for 15 years; Lane 5 non-inoculated tissue kept frozen at −80 °C for 3 months. **b** Detection of TYLCV from crude extracts prepared in 0.5 N NaOH and cleaned as follows: Lane 1 untreated; Lane 2 QIAquick PCR purification column; Lane 3 heated at 65 °C for 10 min; Lane 4 heated at 95 °C for 10 min; Lane 5 amplicon loading buffer contained 5 % SDS; Lane 6 amplicon loading buffer contained 10 % SDS; Lane 7 amplicon loading buffer contained 5 % formamide; Lane 8 amplicon loading buffer contained 15 % formamide. Ten μl of amplified product were loaded into each lane of the 1.5 % agarose gels and stained with ethidium bromide

Amplicons could be clearly visualized in agarose gels after incubation at 65 °C or 95 °C for 10 min, or by the addition of either 5 or 10 % SDS in the loading buffer (Fig. 2b). Use of formamide gave unacceptable results. Amplicons treated with heat or SDS resulted in thicker bands in the agarose gels than those cleaned with PCR purification columns. Both heat and SDS treatment caused some lower molecular weight (LMW) bands to appear at 100–150 bp in gels (Figs. 1 and 2). These LMW bands were not present in those samples cleaned with PCR purification columns (Fig. 2b, lane 2). LMW bands were more obvious in those amplifications using crude extracts (Fig. 2b, lanes 3–8) than purified DNA (Fig. 1). LMW bands did not present a problem for interpretation because the amplicon bands (325–464 bp) were distinctly larger.

### Sensitivity of detection by RPA

The sensitivity of RPA relative to PCR depended upon the source of template DNA. Using purified cloned TYLCV DNA, the detection limit of PCR was estimated to be 9.6 fg of TYLCV DNA while the detection limit of RPA was estimated to be 9.6 pg. RPA was less sensitive than PCR when the template was purified extracts from TYLCV-infected plants (Table 2). RPA generated visible amplicons up to a dilution of 1 × 10^−6^ and 1 × 10^−3^ from DNA extracted from TYLCV-infected tomato and bean leaves respectively. PCR generated a visible amplicon band at dilutions of 1 × 10^−9^ and 1 × 10^−6^ in tomato and bean, respectively, which is 10^4^ and 10^3^ times more sensitive than RPA. However, RPA was more sensitive than PCR when the source of TYLCV DNA was crude extracts (Table 2). PCR was unable to generate a visible amplicon band from any dilution of crude extracts, while RPA produced a visible amplicon band up to a dilution of 1 × 10^−3^ and 1 × 10^−2^ in crude extracts from TYLCV-infected tomato and bean leaves, respectively. RPA successfully generated amplicons from crude extracts of bean and tomato tissues stored at – 20 and −80 °C for several months as well as desiccated tomato tissue stored for 15 year (Fig. 2a).

**Table 2:** Sensitivity of primer pair TYL828F/TYL834R in the detection of *Tomato yellow leaf curl virus* (TYLCV) by recombinase polymerase amplification (RPA) and polymerase chain reaction (PCR) assays

Template source	Tissue source	Detection limit^a^	
		PCR	RPA
Cloned TYLCV genome	--	9.6 × 10 ^−15^ g	9.6 × 10^−12^ g
Purified DNA	TYLCV- infected tomato	1 × 10^−9^	1 × 10^−5^
	TYLCV- infected bean	1 × 10^−6^	1 × 10^−3^
	Non-inoculated tomato	0	0
	Non-inoculated bean	0	0
Crude extraction	TYLCV- infected tomato	0	1 × 10^−3^
	TYLCV- infected bean	0	1 × 10^−2^
	Non-inoculated tomato	0	0
	Non-inoculated bean	0	0

^a^ Detection Limit – the last dilution in which an amplicon could be visually detected by ethidium staining in agarose gel electrophoresis

Similar results were observed in the detection of BGYMV and ToMoV by RPA amplification using crude extracts. Both BGYMV and ToMoV could be detected up to a 1 × 10^−3^ dilution of crude extracts of bean leaf tissue (data not shown). PCR was unable to amplify either BGYMV or ToMoV in crude extracts.

### Downstream applications of RPA

Amplicons generated by RPA from purified DNA and treated after amplification with PCR purification columns were similar to PCR-generated amplicons in their suitability for direct sequencing, digestion with *Cla*I and TA cloning with 100 % efficiency (Table 3). RPA products generated using crude extract templates required different handling for some of the downstream applications (Table 3). RPA products from crude extracts could be cloned if the amplified DNA was cleaned with QIAquick PCR purification columns. RPA amplicons reactions denatured at 65 °C for 10 min could be digested with *Cla*I or cloned if centrifuged at 3800 rcf for 20 s before restriction digestion or cloning.

**Table 3:** Effect of type of template and cleaning method on the usefulness of amplicons generated by recombinase polymerase amplification (RPA) of *Tomato yellow leaf curl virus* using primer pair TYL828F/TYL834R in three downstream applications

Template	Amplicon treatment	TA Cloning efficiency (%)^a^	Restriction endonuclease digestion	Direct sequencing
Purified DNA	No treatment	0	yes	nt^b^
	denatured 65 °C	0	no	nt
	denatured 65 °C then centrifuged	80	yes	2/2
	PCR purification column	100	yes	1/2
Crude extraction	No treatment	0	yes	nt
	denatured 65 °C	0	no	nt
	denatured 65 °C then centrifuged	0	yes	2/2
	PCR purification column	100	yes	2/2

^a^ Cloning efficiency is defined as the number of colonies with the expected insert in ten randomly selected white colonies

^b^ nt = Not tested

### Clinical trial

**Fig. 3:**
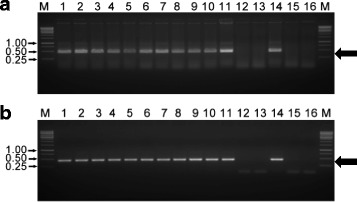
Clinical trial comparing RPA and PCR for the detection of *Tomato yellow leaf curl virus* (TYLCV) in field samples. Assays used primer pair TYL828F/TYL834R for the detection of TYLCV in samples collected from thirteen plants from two tomato field sites. **a** Amplification using RPA and **b** Amplification using PCR. Samples 1 through 10 had symptoms characteristic of an established infection of TYLCV while the symptoms in samples 11–13 were less characteristic. Lane M: 50 bp ladder MW standard, size is indicated in kilobases (kb); Lanes 1 – 13 samples 1 – 13; Lane 14 TYLCV-infected plant sample; Lane15 non-inoculated tomato; Lane 15 water control

Expected size amplicons were generated from 11 of the 13 field samples using RCA and primer pair TYL828F/TYL834R and results were identical in the two replications (Fig. 3). These results were identical to those obtained with PCR using the same primer pair. Ten samples, which had symptoms consistent with those of TYLCV, tested positive for TYLCV by both assays. Three samples, which had symptoms that could be interpreted as possible early symptoms of TYLCV, yielded identical results using the two assays; one sample tested positive and the other two tested negative.

### Comparison of cost and time

The costs of extraction and amplification at the UF Plant Diagnostic Clinic varied with the extraction method, the assay selected, the type of template and the number of samples processed (Additional file 2: Table S1). The combination of RPA and a crude extraction method was similar in cost of reagents to that of PCR. The cost to amplify by PCR was lower than that of RPA, but the total reagent cost was higher because of the dependence of PCR upon relatively purified DNA. The higher reagent costs of amplification by RPA were offset by the ability of RPA to amplify targets using crude extracts.

RPA always required less time than PCR regardless of the purity of the template (Additional file 2: Table S1). The assay that required the least amount of time was amplification by RPA using crude extracts, which required less than one hour (0.68 h) compared to that of PCR (2.5 h). This was due to differences in time required for extraction as well as amplification.

When time was factored into costs, RPA using crude extracts was the least expensive assay (Additional file 2: Table S1). Using $35.00 as an estimate for 1 h of labor, the cost of detection for single samples using PCR was much higher than that of RPA using crude extracts or purified DNA. The number of samples processed at the same time had some effect on costs. Thirteen samples processed by RPA using crude extracts was the least expensive assay based on costs of reagents plus labor. The most expensive assay was RPA using purified DNA followed by PCR using the same type of template.

## Discussion

These results is research demonstrate that RPA can specifically detect three begomoviruses (BGYMV, ToMoV and TYLCV). RPA was able to detect as little as 9.6 × 10^−12^ g of TYLCV DNA, which was 1000 fold less than that of PCR in the absence of plant contaminants. RPA was able to detect 9.6 pg and PCR was able to detect 9.6 fg of purified TYLCV DNA. In the presence of plant contaminants, PCR was unable to generate visible amplicons, while RPA was able to generate amplicons but with a reduction in sensitivity. However, this was still within an acceptable range as it was equivalent to or greater than that of commonly used diagnostic assays such as ELISA and lateral flow immunochromatographic assays [33]. Our results in the sensitivity of detection of TYLCV DNA using RPA were similar to those reported for transcripts of two RNA plant viruses [18, 19]. In addition, RPA was comparable to PCR in specificity and flexibility for use in downstream applications. Amplicons generated by RPA were readily TA cloned, digested with an endonuclease restriction enzyme, and sequenced directly, as with amplicons generated by PCR, but unlike some other isothermal reactions such as LAMP.

While the manufacturer recommends removal of DNA-binding proteins from RPA-generated amplicons using PCR purification columns before visualization by electrophoresis, this increases the time and expense of the assay. Results from this study indicate that less expensive and faster methods, such as the use of heat or the addition of SDS, could be used to achieve acceptable results. In addition, the cost and time required to conduct an RPA assay could be reduced by the use of a crude extraction method for the template. Use of these modifications demonstrated that RPA can be used as an assay for the detection of plant pathogens for the same cost and in less time than PCR with adequate sensitivity, and without sacrificing specificity or flexibility for use in downstream applications.

The detection of TYLCV by RPA was comparable to sensitivity, specificity, independence from thermocyclers and speed with results reported with the use of LAMP, another isothermal assay and was superior with respect to downstream applications [26, 28]. RPA offers an assay speed similar to that of qPCR without the need to purchase and maintain real-time amplification instruments [1, 35, 36]. RPA represents a significant improvement in molecular diagnostic tools, with excellent potential for adoption in diagnostic service laboratories worldwide, especially those with minimal infrastructure.

RPA is a versatile molecular diagnostic tool that extends the capabilities of diagnostic facilities without access to a thermocycler. Pathogen specific assays can easily be developed to detect plant viruses using RPA with only minor changes to primers originally developed for use in PCR [13, 37, 38]. In addition, RPA can be modified for detection in micro fluid and solid phase based devices [14, 18, 19, 39], and with the addition of a reverse transcription step, can be used with RNA templates [13, 15, 18, 19].

## Conclusions

RPA has been used to detect a number of human and animal pathogens, however few RPA assays have been reported for the detection of plant pathogens [18, 19]. The results of these studies using begomoviruses demonstrate that RPA offers significant advantages over current PCR technologies for the detection of plant viruses by diagnostic laboratories. RPA was less expensive and faster than PCR. While RPA was not as sensitive as PCR, it was equivalent to the sensitivity of currently used diagnostic techniques such as ELISA and lateral flow immunochromatographic assays. However, RPA was superior to these protein based assays in its ability to support downstream applications such as restriction enzyme digestion, TA cloning and direct sequencing. The speed, cost, specificity, sensitivity and ease of use make RPA an ideal assay for plant disease diagnostic laboratories.

## Additional files

Additional file 1: Figure S1. Recombinase polymerase amplification RPA detection of two begomoviruses. Primer pairs, BGY1141F/BGY1142R and ToMo1131F/ToMo1139R were used for the detection of (A) *Bean golden yellow mosaic virus* (BGYMV) and (B) *Tomato mottle virus* (ToMoV) respectively. Template was purified DNA and amplicons were cleaned by heating to 65 °C for 10 min. Contents of lanes for both (A) and (B): Lane M: 50 bp ladder MW standard, size is indicated in kilobases (kb); Lane 1 non-inoculated tomato; Lane 2 non-inoculated bean; Lane 3 BGYMV-infected bean; Lane 4 *Euphorbia mosaic virus* (EuMV)-infected bean; Lane 5 *Sida golden mottle virus* (SiGMoV)-infected bean; Lane 6 ToMoV-infected bean; Lane 7 *Tomato yellow leaf curl virus* (TYLCV)-infected bean; Lane 8 water control. Ten μl of amplified product were loaded into each lane of the 1.5 % agarose gels and stained with ethidium bromide. (PDF 242 kb) Additional file 2: Table S1. Assay costs for recombinase polymerase amplification (RPA) and polymerase chain reaction (PCR) assays for detection of TYLCV. (PDF 39 kb)
