# Supplementary figures and images for: Evaluation of recombinase polymerase amplification for detection of begomoviruses by plant diagnostic clinics
Source: Virol J. 2016 Mar 22;13:48. doi: 10.1186/s12985-016-0504-8 (PMC4802622; doi:10.1186/s12985-016-0504-8)

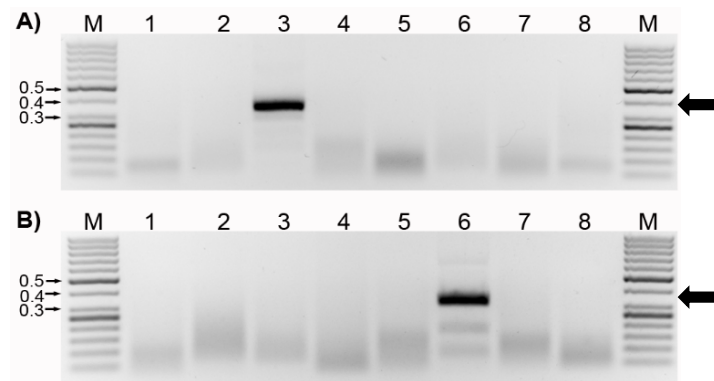

Additional file 1  
Figure S1

Supplement: Additional file 1: Figure S1. — Recombinase polymerase amplification RPA detection of two begomoviruses. Primer pairs, BGY1141F/BGY1142R and ToMo1131F/ToMo1139R were used for the detection of (A) Bean golden yellow mosaic virus (BGYMV) and (B) Tomato mottle virus (ToMoV) respectively. Template was purified DNA and amplicons were cleaned by heating to 65 °C for 10 min. Contents of lanes for both (A) and (B): Lane M: 50 bp ladder MW standard, size is indicated in kilobases (kb); Lane 1 non-inoculated tomato; Lane 2 non-inoculated bean; Lane 3 BGYMV-infected bean; Lane 4 Euphorbia mosaic virus (EuMV)-infected bean; Lane 5 Sida golden mottle virus (SiGMoV)-infected bean; Lane 6 ToMoV-infected bean; Lane 7 Tomato yellow leaf curl virus (TYLCV)-infected bean; Lane 8 water control. Ten μl of amplified product were loaded into each lane of the 1.5 % agarose gels and stained with ethidium bromide. (PDF 242 kb) [file 12985_2016_504_MOESM1_ESM.pdf]
