# Supplementary material for: Evaluation of recombinase polymerase amplification for detection of begomoviruses by plant diagnostic clinics
Source: Virol J. 2016 Mar 22;13:48. doi: 10.1186/s12985-016-0504-8 (PMC4802622; doi:10.1186/s12985-016-0504-8)
Supplement: Additional file 2: Table S1. — Assay costs for recombinase polymerase amplification (RPA) and polymerase chain reaction (PCR) assays for detection of TYLCV. (PDF 39 kb) [file 12985_2016_504_MOESM2_ESM.pdf]

Additional file 2

Table S1. Assay costs for recombinase polymerase amplification (RPA) and polymerase chain reaction (PCR) assays for detection of TYLCV

Assay	Template	Processing Sample Size[a]	Extraction		Amplification[c]		Total Cost Reagents ($)	Total Time (h)	Total Costs ($)[d]
			Reagent cost ($)[b]	Time (h)	Reagent cost ($)	Time (h)			
PCR	Pur DNA	1	4.26	1.00	1.32	1.50	5.40	2.50	92.90
RPA	Pur DNA	1	4.26	1.00	5.50	0.67	9.76	1.67	68.21
RPA	Crude extract	1	0.00	0.01	5.50	0.67	5.50	0.68	29.30
PCR	Pur DNA	13	55.38	1.20	17.16	1.83	72.54	3.03	178.59
RPA	Pur DNA	13	55.38	1.20	71.50	0.75	126.88	1.95	195.13
RPA	Crude extract	13	0.0	0.17	71.50	0.75	71.50	0.92	103.73

[a] Number of plant samples tested at one time

[b] Costs of reagents per sample used in these studies. All costs are estimated from prices current as of June 2015 and are in US dollars.

[c] For PCR assumes 1.3 h amplification program; for RPA assumes 20 min amplification, heating at 65 ^{o}C for 10 min followed by centrifugation at 3,800 rcf for 10 min.

[d] Reagent and labor costs: These are the costs incurred in the reported studies. Costs do not include agarose gel electrophoresis. A rate of $35.00 per hour for labor was used in the calculations.
